# EphH, a unique epoxide hydrolase encoded by Rv3338 is involved in the survival of *Mycobacterium tuberculosis* under *in vitro* stress and vacuolar pH-induced changes

**DOI:** 10.3389/fmicb.2022.1092131

**Published:** 2023-01-26

**Authors:** Tanu Garg, Swetarka Das, Shriya Singh, Mohmmad Imran, Atri Mukhopadhyay, Umesh D. Gupta, Sidharth Chopra, Arunava Dasgupta

**Affiliations:** ^1^Molecular Microbiology and Immunology Division, CSIR-Central Drug Research Institute, Lucknow, India; ^2^Academy of Scientific and Innovative Research (AcSIR), Ghaziabad, India; ^3^National JALMA Institute for Leprosy and Other Mycobacterial Diseases, Agra, India

**Keywords:** *Mycobacterium tuberculosis*, epoxide hydrolase, acid stress, vacuolar pH, stress response

## Abstract

**Introduction:**

*Mycobacterium tuberculosis* (Mtb), one of the deadliest human pathogen, has evolved with different strategies of survival inside the host, leading to a chronic state of infection. Phagosomally residing Mtb encounters a variety of stresses, including increasing acidic pH. To better understand the host-pathogen interaction, it is imperative to identify the role of various genes involved in the survivability of Mtb during acidic pH environment.

**Methods:**

Bio-informatic and enzymatic analysis were used to identify Mtb gene, Rv3338, as epoxide hydrolase. Subsequently, CRISPRi knockdown strategy was used to decipher its role for Mtb survival during acidic stress, nutrient starvation and inside macrophages. Confocal microscopy was used to analyse its role in subverting phagosomal acidification within macrophage.

**Results:**

The present work describes the characterization of Rv3338 which was previously known to be associated with the aprABC locus induced while encountering acidic stress within the macrophage. Bio-informatic analysis demonstrated its similarity to epoxide hydrolase, which was confirmed by enzymatic assays, thus, renamed EphH. Subsequently, we have deciphered its indispensable role for Mtb in protection from acidic stress by using the CRISPRi knockdown strategy. Our data demonstrated the pH dependent role of EphH for the survival of Mtb during nutrient starvation and in conferring resistance against elevated endogenous ROS levels during stress environment.

**Conclusion:**

To the best of our knowledge, this is the first report of an EH of Mtb as a crucial protein for bacterial fitness inside the host, a phenomenon central to its pathogenesis.

## Introduction

*Mycobacterium tuberculosis*, the etiologic agent of tuberculosis (TB), is responsible for causing 2,590,000 cases,1.6 million deaths and 2.2 million new cases globally by 2021 (World Health Organization, 2022). This enormous success is attributed to its ability to survive within the host by overcoming a functional immune response. Macrophages are the primary reservoir of Mtb for its growth, survival and persistence. After being phagocytosed, Mtb encounters a variety of toxic stresses, such as an increasingly acidic environment in different compartments. As a successful macrophage colonizer, it has adapted to overcome the hostile environment by arresting phagosome maturation, thus multiplying within a hospitable phagosome (Schnappinger et al., 2003; Ehrt and Schnappinger, 2009; Rohde et al., 2012). Several studies have deciphered mechanisms by which pathogenic mycobacteria can successfully prevent phagolysosomal fusion and replicate in a growth-tolerant vacuolar compartment having acidic pH of 6.2 (Armstrong and Hart, 1971; Russell, 2001; Rohde et al., 2012). Mtb-containing vacuole is characterized by incomplete acidification due to limited accumulation of vacuolar ATPases as well as failure to acquire lysosomal GTPase Rab7 and mature lysosomal hydrolases (Russell, 2001). As reported earlier, pathogenic mycobacteria that arrest maturation reside in a resting phagosome having an intracellular pH of 6.2, whereas, in the activated phagosome, pH drops to 5.2 (Armstrong and Hart, 1971; Hart and Armstrong, 1974; Schaible et al., 1998; MacMicking et al., 2003). Intriguingly even when Mtb is exposed *in vitro* to a variety of acidic pH levels (from 6.2 to 4.5), comparable with the acidic phagosomal milieu of resting or active phagosomes, it perpetuates a neutral intrabacterial pH of 7.2 (Via et al., 1998; Vandal et al., 2008, 2009) demonstrating that during infection Mtb maintains intrabacterial pH homeostasis. In this context, acidic pH is one of the vital intraphagosomal signals recognized by Mtb to modulate the metabolism of its genome to overcome stress and establish a chronic infection (Schnappinger et al., 2003). Therefore, the mechanisms by which Mtb senses the phagosomal environment and modulates its gene expression profile are of significant interest as they aid Mtb in surviving in this hostile situation.

Previous studies reported that aprABC, one of the Mtb-specific locus among the “first responders,” was induced after 2 h post phagocytosis and required for adaptation inside macrophage phagosome (Vandal et al., 2008). The *aprABC* operon consists of three genes designated as *aprA* (Rv2395A), *aprB* (Rv2395B), and *aprC* (Rv2396), respectively, and several other Mtb genes show differential expression when the locus is activated while encountering acidic pH. The differential gene expression was confirmed by microarray analysis (Abramovitch et al., 2011). The study had shown that the deletion of *aprABC* hampers the gene expression, affecting intracellular growth, aggregation, and relative levels of the storage as well as cell wall lipids (Abramovitch et al., 2011). Rv3338 is a conserved hypothetical protein associated with this aprABC locus and is predicted as a member of the alpha/beta hydrolases superfamily. Rv3338 is included in the list of aprA-induced genes prepared through microarray analysis. The study has reported approximately 2-fold induction of aprA and aprB transcripts at pH 6.0 and pH5.5. The microarray analysis was conducted to study the expression patterns of aprA, aprB, and aprC-induced genes at acidic pH. Perchance, Rv3338 was included in the most prominent group of aprA-induced genes, including a cluster of 21 predicted cistrons spanning a 40.4 kb region between Rv3301c and Rv3338. In this region, 20 genes were significantly repressed in the knockout mutant of aprABC compared to the knockout mutant of aprBC and aprC. This means these play a vital role in the survival of Mtb during acid stress *in vitro* and inside macrophages. The homologues of this gene have been reported to be present in *M. bovis*, *M. marinum*, *M. leprae*, and *M. smegmatis*. Conserved hypothetical proteins like Rv3338 constitute almost 25% of 4,000 ORFs of Mtb (Cole et al., 1998) and are reported to be involved in various functions ranging from drug–resistance to host-pathogen interactions. In the context of better understanding the Mtb biology and its interaction with the host, characterization of this enormous albeit overlooked pool of ORFs is essential (Doerks et al., 2012; Mazandu and Mulder, 2012; Yang et al., 2019).

A bioinformatic analysis of Rv3338 in the Uniprot and ESTHER database collection revealed that it has an AB hydrolase-1 domain and could be a potential epoxide hydrolase (EH). This class of enzymes binds to specific epoxides and changes them to corresponding diols. They are present in all living organisms and play important roles, including detoxification, lipid metabolism and signaling (Biswal et al., 2008; Cirillo et al., 2009; Schulz et al., 2020). Eight different EH enzymes (EphA-G, MesT) have been reported in the Mtb genome. Some of them are proposed as potential therapeutic targets due to their role in detoxification (Biswal et al., 2008; Cirillo et al., 2009; Schulz et al., 2020). In this manuscript, we identified and characterized a EH from Mtb encoded by Rv3338 and renamed it EphH. We demonstrated the importance of EphH in the survival of the bacilli under *in vitro* acidic stress and its pH dependent role during nutrient-deprived conditions as well as its *ex vivo* role in subverting phagosomal acidification.

## Materials and methods

### Bacterial strains, cell lines, and reagents

*Escherichia coli* DH5α and BL21 (DE3) Rosetta were grown in Luria–Bertani (LB) broth (Becton Dickinson, Franklin Lakes, NJ, United States) supplemented with Kanamycin (50 μg ml^−1^), and chloramphenicol (34 μg ml^−1^) whenever required. Mtb was cultured in Middlebrook (MB) 7H9 broth medium (Becton Dickinson, Franklin Lakes, NJ, United States) supplemented with 0.2% glycerol, 0.5 g L^−1^ FAF-BSA, 0.085% NaCl, 0.05% Tween-80 and MB 7H11 agar medium (supplemented with 0.2% glycerol, 0.5 g L^−1^ FAF-BSA, 0.085% NaCl); 100 ng ml^−1^ Anhydrotetracycline (ATc) and 25 μg ml^−1^ kanamycin were used wherever required.

Vacuolar H^+^-ATPase (V-ATPase) antibody was procured from Santa Cruz Biotechnology, TX, United States. Lysotracker DND red99, Alexa Fluor 594 conjugated secondary antibody and Concanamycin-A (CCA) were purchased from Thermo Fisher Scientific, MA, United States. Fluorescein 5(6)-isothiocyanate (FITC) was bought from Sigma-Aldrich, St. Louis, MO, United States. Mouse macrophage cell line J774A.1 was procured from ATCC (Manassas, VA, United States) and maintained in RPMI 1640 (Gibco, Thermo Fisher Scientific, MA, United States) supplemented with 200 mM glutamine, 50 μg ml^−1^ of neomycin, 10% fetal bovine serum (Gibco, Thermo Fisher Scientific, MA, United States) at 37°C in the presence of 5% CO_2_. Supplementary Tables S1–S3 contains all primers used in this study.

### Overexpression and purification of recombinant EphH

Mtb H37Rv genomic DNA was used as a template to amplify the 645 bp EphH. The amplicon was cloned into expression vector pET28a using asymmetric NdeI and XhoI restriction sites. For overexpression of recombinant EphH, *E. coli* BL21 (DE3) Rosetta strain was used. Cells harboring EphH-pET28a were grown to an OD_600_ of 0.6. For recombinant protein induction, Isopropyl β-D-1-thiogalactopyranoside (IPTG) was added to a final concentration of 0.1 mM and growth was continued at 37°C with shaking for 3 h. Cells were lysed, and His tagged EphH was purified from cell lysates by Ni^2+^-NTA chromatography (Supplementary Figure S1).

For purification, 1 L of culture was induced with 0.1 mM IPTG and grown for 3 h at 37°C with continued shaking. The supernatant was subjected to a pre-equilibrated Ni^2+^-NTA column incubated at 4°C. After incubation, it was washed serially with two washes of wash buffer WB (wash buffer) 1 [50 mM Tris-HCl (pH 8.0), 200 mM NaCl, 30 mM Imidazole, and 5% glycerol], WB 2 [50 mM Tris-HCl (pH 8.0), 200 mM NaCl, 50 mM Imidazole, and 5% glycerol] and one wash of WB 3 [50 mM Tris-HCl (pH 8.0), 200 mM NaCl, 100 mM Imidazole, and 5% glycerol]. Subsequently, the purified recombinant protein was eluted with elution buffer [50 mM Tris-HCl (pH 8.0), 200 mM NaCl, 300 mM Imidazole, and 5% glycerol]. The protein was dialyzed in three MWCO membrane (Thermo Fisher Scientific, MA) against 500 ml dialysis buffer [50 mM Tris-HCl (pH 8.0), 200 mM NaCl, and 5% glycerol] at 4°C. The purified recombinant EphH (rEphH) concentration was determined by Bradford colorimetric assay (HiMedia Laboratories, Mumbai, India).

### Circular dichroism analysis

The purified rEphH was further subjected to spectroscopy analysis to study the proper folding of the protein. Using a CD spectrophotometer (J815, JASCO, Tokyo, Japan), a far UV-CD spectrum of EphH protein was measured. In a 2 mm path length cuvette at room temperature, the spectrum of EphH was recorded at a wavelength ranging from 195 to 250 nm at a scan speed of 10 nm/min at 25°C. The range of 5–20 μM protein dialyzed against phosphate buffer pH 8.0 containing 50 mM NaCl was used for measuring the spectra. The normalization of obtained values was done by subtracting the baseline recorded for the buffer. The molar ellipticity recorded by the CD spectrophotometer was expressed as ([θ], degcm^2^ dmol^−1^).

### EH activity assay

I. *Using PHOME as a substrate*: The enzymatic activity was determined using (3-phenyl-oxiranyl)-acetic acid cyano-(6-methoxynaphthalen-2-yl)-methyl ester (PHOME), a specific fluorescent substrate for epoxide hydrolases, according to the protocol described in earlier reports (Wolf et al., 2006). Briefly, 50 μM of PHOME was incubated with different concentrations (30, 60, and 90 nM) of purified EphH in 25 mM BisTris–HCl buffer (pH 7.0) containing 0.1 mg ml^−1^ BSA to ensure linear substrate hydrolysis over a 1 h period with different concentrations of enzyme. Fluorescence intensity was monitored every 10 min for 1 h by Spectra Fluor Plus fluorescent plate reader (Tecan Systems, San Jose, CA, United States) using the following settings: excitation wavelength: 330 nm (bandwidth, 20 nm); emission wavelength: 465 nm (bandwidth, 20 nm); manual gain: 60; integration time: 40 μs; number of flashes: 3. Michaelis–Menten parameters were calculated after that with the GraphPad Prism version 8.02 (San Diego, CA, United States). The enzyme activity was also determined with its specific inhibitor (12-(3-adamantane-1-yl-ureido)-dodecanoic acid-AUDA) and commercially available human recombinant sEH as a positive control.
II. *Using styrene oxide as a substrate*: The commercial substrate used in the above study is sensitive to esterase activity. Therefore, activity of EphH was also confirmed by colorimetric “Red assay” using styrene oxide as the substrate according to the described protocol (Chownk et al., 2017). The concentration range of EphH used for assay was 10–60 ng/μl; no enzyme group was used as the control. The enzyme solution was diluted up to 40 μl and was mixed with 50 μl of 20 mM sodium phosphate buffer (pH 7.5). 10 μl of 100 mM styrene oxide racemic mixture in 20% acetonitrile was used to start the reaction. The reaction was stopped after 15 min at 30°C by adding 50 μl of 5 mM NaIO_4_ in 90% (v/v) acetonitrile. The remaining NaIO_4_ was titrated after 1 h at room temperature by adding 50 μl of 6 mM adrenaline in 10% (v/v) acetonitrile. The absorbance was read at 490 nm after 15 min.

### Antibody generation

Polyclonal antibody against EphH was raised at National Laboratory Animal Center (NLAC), CSIR-Central Drug Research Institute, Lucknow, India, according to the approved protocol of Institutional Animal Ethics Committee (Approval reference No. IAEC/2019/142/Renew-0/Dated-30/08/2019) using 15 weeks old NZW rabbit. Using a glass syringe linked to a three-way stopcock, 500 μg purified EphH at a concentration of 1 mg ml^−1^ was emulsified in a 1:1 ratio with Freund’s incomplete adjuvant and injected subcutaneously. Four booster immunizations were given at 2-week intervals to achieve the highest serum antibody titre. The specificity and purity of obtained antisera were checked with whole cell lysate of Mtb (Supplementary Figure S4).

### Construction of EphH knockdown (KD) strain

The EphH KD strain was constructed employing clustered regularly interspaced short palindromic repeats interference (CRISPRi) approach as described earlier using pLJR965 vector. The CRISPRi technology used in this study is a robust, easily engineered, and scalable platform for regulated gene silencing. In contrast to other CRISPRi systems based on Streptococcus pyogenes Cas9, this system based on *Streptococcus thermophilus* deactivated Cas9 typically achieves 20–100-fold knockdown of endogenous gene expression with minimal proteotoxicity. The CRISPRi model system used for generating EphH knockdown is based on optimized Anhydrotetracycline (ATc) regulated promoters. The CRISPRi-mediated transcriptional repression is ATc-inducible (P_Tet_) which directs dcas9 to specific DNA targets by ATc-inducible or constitutively (P_con_) expressed sgRNA, which prevents transcription initiation or elongation. A Protospacer adjacent motif (PAM) sequence is required to recognize the DNA region targeted for cleavage. In the study 5′-NNAGAAT-3′ PAM sequence was used for sgRNA design. The addition of Atc leads to knockdown of the desired gene (Rock et al., 2017). The clone was electroporated in Mtb, and the colonies were selected on MB 7H11 kanamycin plates (supplemented with 0.2% glycerol, 0.5 g L^−1^ FAF-BSA, 0.85% NaCl).

### RNA extraction and quantitative RT-PCR

RNeasy mini kit (Qiagen, Hilden, Germany) was used for total RNA extraction from Mtb cultures according to standard protocol (Rastogi et al., 2016). The reverse transcription of about 1 μg of the total RNA sample was done in a 20 μl reaction using Go Script™ Reverse Transcriptase cDNA Synthesis Kit (Promega, WI, United States). The synthesized cDNA was used directly for qRT-PCR. Primers used are listed in Supplementary Table S3. The specificity of the PCR products was checked by melting curve analysis. SigA gene was used as endogenous control, and results were normalized using the 2^−ΔΔ*C*T^ method (Livak and Schmittgen, 2001).

### *In vitro* stresses

- Acidic stress

For examining the effect of acidic stress, WT and EphH KD strains were grown up to mid-log phase (OD_600_ 0.4–0.5) and exposed to 7H9 medium adjusted to a range of 7.0, 6.0, and 5.5 using Hydrochloric acid (HCl) and buffered using 100 mM MES (Mehta et al., 2016). The cultures were adjusted to an OD_600_ of 0.05 from day 0. The transcript levels and survival were monitored after each day. The growth curves for different pH were also analyzed (Supplementary Figures S6–S8).

II. Nutrient depletion condition

For analyzing the influence of nutrient depletion, the WT and EphH KD strains were grown up to mid-log phase (OD_600_ 0.4–0.5) and were washed two times with PBS and adjusted to an OD_600_ of 0.05, exposed to PBS+ 0.05% tween-80 for 14 days at pH 7.0, 6.0, and 5.5. In this case, the survival and relative expression levels were monitored every 2nd day.

### Intracellular survival studies

Murine macrophage cells J774A.1 (10^5^) were seeded in 24 well plates and infected with WT and KD strains at a multiplicity of infection (MOI) of 10. The bacteria were allowed to internalize for 4 h. For CCA experiments, 10 nM CCA was used to treat J774A.1 cells with control group acetonitrile (vehicle control) 1 h before infection (Sullivan et al., 2012; Mehta et al., 2016). After infection, the cells were washed with PBS and resuspended in RPMI medium containing CCA as needed. The cells were maintained in medium containing CCA throughout the experiment. At indicated time points, the infected cells were lysed using PBS+ 0.06% SDS serially diluted in Tyloxapol Normal Saline (TNS), plated on 7H11 medium at 12, 24, 36 h post-infection and incubated at 37°C for 3 weeks. The experiments were done in triplicate.

### ROS analysis

Endogenous ROS levels were analyzed during acidic and nutrient depletion conditions. The WT & EphH KD were grown to an OD_600_ of 0.4–0.5 and OD_600_was adjusted to 0.05 and was exposed to 7H9 medium adjusted at pH 7.0, pH 6.0, pH 5.5, respectively. For nutrient-depleted conditions the WT and EphH KD strains were grown up to mid-log phase (OD_600_ 0.4–0.5) and were washed two times with PBS and adjusted to an OD_600_ of 0.05, exposed to PBS+ 0.05% tween-80. The ROS levels were monitored after every 24 h for acidic stress and every 48 h for nutrient stress. At each time point, bacteria were spun down and resuspended in 1 ml of the same medium before OD_600_ measurement. OD_600_ were adjusted to OD = 0.1 with the media, and 200 μl bacteria were added to a 96-well plate (black plate/clear bottom). OD_600_ was recorded for future normalization. To measure the amount of ROS generated, Cell ROX Green Reagent (Thermo Fisher Scientific MA, United States) – a novel fluorogenic probe for measuring oxidative stress in live cells was used, with a final concentration of 5 μM and incubated for 2 h at 37°C. Fluorescence was recorded with spectra Fluor Plus fluorescent plate reader (Tecan Systems, San Jose, CA, United States; excitation 485 nm/emission 520 nm). Fluorescence measurements were normalized to OD_600_ (Fluorescence/OD_600_) and expressed as arbitrary units (A.U.) excitation 485 nm/emission 520 nm. Each condition was recorded in triplicate, and the mean was calculated.

### Confocal microscopy

J774A.1 (0.3 × 10^6^) cells were seeded and infected with WT, and EphH KD strains at an MOI of 10 for 4 h with Logarithmically grown Mtb strains stained with FITC as described (Rohde et al., 2007; Sullivan et al., 2012; Mehta et al., 2016). One hour before the time point, the medium was substituted with complete fresh RPMI containing 100 nM Lysotracker red DND-99 (Mehta et al., 2016). The immunostaining procedure for V-ATPase was done according to the published protocol (Mehta et al., 2016). Briefly, cells were fixed for 15 min at room temperature (RT) in 4% (v/v) paraformaldehyde in 1×PBS, followed by three washes with 1×PBS and permeabilization by 0.2% (v/v) Triton X-100 in 1×PBS for 10 min at RT. The cells were blocked for 30 min at RT with 3% (w/v) BSA and 0.5% (v/v) Tween 80 in PBS and incubated at RT with the appropriate dilution of anti-V-ATPase antibody in BSA-PBS. The slides were washed thoroughly with 1xPBS and then incubated with a 1:1,000 dilution of Donkey anti-mouse Alexa 594-conjugated IgG for 45 min at room temperature. Finally, samples were mounted with ProLong antifade reagent (Thermo Fisher Scientific, MA, The United States) and examined under a Leica TCS SP5 confocal microscope. *z* stacks were recorded using 63× oil immersion objective, subsided into two-dimensional images and analyzed by LAS AF version 4.0, build 7266. A minimum of 50 phagosomes were observed for all of the phagosomal markers in around five captured fields, leading to an estimated analysis of 100–200 bacteria/well. At least three replicates were scored for each experimental group.

### Data analysis

GraphPad Prism version 8.02 (San Diego, CA, United States) was used for data analysis. The significance of analyzed differences was calculated with one-way or two-way ANOVA with Bonferroni’s post-tests. The statistically significant differences were those having *p* values <0.05, <0.01, or <0.001, denoted by *, **, or ***, respectively.

## Results

### Predicted bioinformatics analysis shows the similarity of EphH to various Mtb EHs

**Figure 1:**
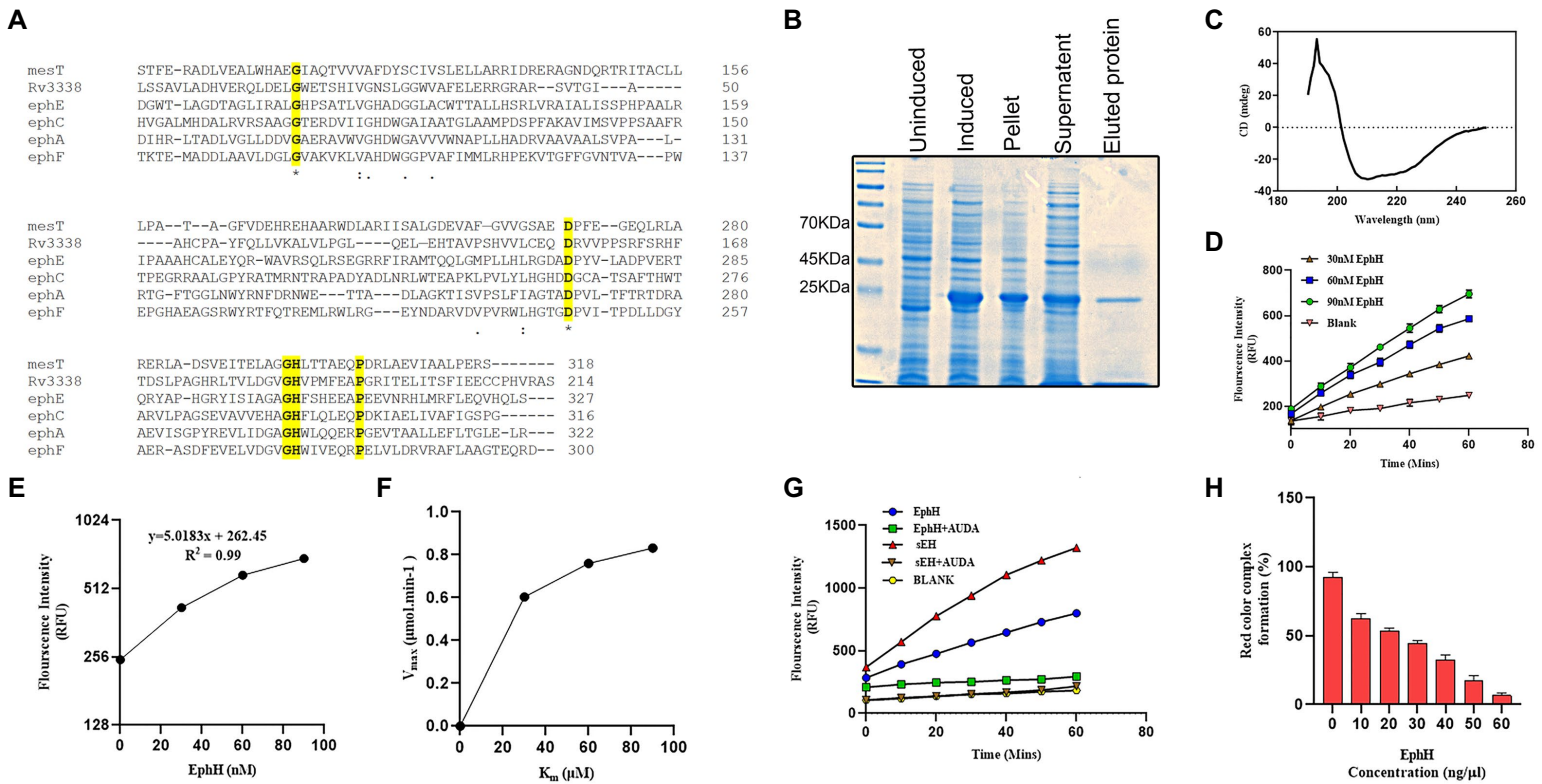
Predicted bioinformatics analysis shows the similarity of Rv3338 to epoxide hydrolase, and its recombinant protein purified from *Escherichia coli* shows potent epoxide hydrolase activity. **(A)** The CLUSTAL omega (1.2.4) multiple sequence alignment results of various Mtb epoxide hydrolases with Rv3338. The identical *active site 1 shown is the site which plays an orientation role for the imidazole group of His-298. The identical *active site 2 is the proton acceptor site. **(B)** SDS-PAGE of His-tagged Rv3338 (23.2 kDa) recombinant protein purified by Ni-nitrilotriacetic acid (Ni^+2^-NTA) affinity chromatography. **(C)** Shows refolding of the recombinant protein to a stable native structure confirmed by far-UV circular dichroism **(D)** Epoxide hydrolase activity of EphH was tested using different concentrations of the recombinant enzyme with 50 μM substrate concentration for 1 h. The graph shows the linear hydrolysis of the substrate over a 1 h time period through the fluorescence released. **(E)** Graph showing the *R*^2^ value of the previous graph. **(F)** Represent Micheles-Menten graph of **(D)** showing enzyme-substrate kinetics. **(G)** Shows the EphH enzyme activity (concentration of EphH-30 nM) with its specific inhibitor (AUDA) and positive control (sEH; concentration 30 nM). **(H)** Shows the epoxide hydrolase activity of EphH with styrene oxide. All the data plotted are mean ± SD of three independent experiments.

To get into structural insights of EphH, bioinformatic approaches were used. A search using the ESTHER database revealed that EphH contains AB hydrolase-1-domain and belongs to the alpha/beta hydrolases superfamily. Furthermore, to find out its similarity with other Mtb EHs, multiple sequence alignment with amino acid sequences was performed on CLUSTAL-Ʊ. This result showed that EphH has two identical active sites and conserved residues with other Mtb EHs-MesT, ephA, ephC, ephE, and ephF (Figure 1A).

### Purified, rEphH protein shows EH activity

To gain the first insight into the biochemical activity of EphH, *in vitro* assays were set up to carry out its EH activity, as predicted by the bioinformatics data. The recombinant EphH was purified through Ni-Nta^+2^ chromatography (Figure 1B), and the folding of native recombinant protein was checked with CD analysis (Figure 1C). The CD data was analyzed using K2D3 software, which shows the presence of both α-helix (63.93%) and β-sheet (5.19%; Supplementary Figure S2).

i. *EH activity with PHOME*: The purified rEphH was further used to conduct enzymatic analysis. The assay was performed using PHOME (3-phenyl-cyano (6-methoxy-2-naphthalenyl) methyl ester-2-oxiraneacetic acid), a specific fluorogenic substrate for EH activity at different concentrations of EphH (30, 60, and 90 nM) in 25 mM BisTris–HCl buffer (pH 7.0) containing 0.1 mg ml^−1^ BSA. The results demonstrated that EphH exhibits significant hydrolysis of PHOME, confirming its role as an EH (Figure 1D). The correlation between the RFU and rEphH concentration was determined as 0.99 (*R*^2^ value; Figure 1E). The enzyme kinetics were determined using Michaelis–Menten kinetic parameters. For that experiment, the assay reaction mixture was incubated for 1 h, and reading was taken every 10 min (Figure 1F) and found *K*_m_, *V*_max_, and *K*_cat_ values of 21.72 μM, 1.027 μmol.min^−1^, and 18.45 s, respectively (Table 1). The kinetic parameters of EphH were at a level comparable to sEH. Additionally, significant inhibition of EphH activity by AUDA (12-[[(tricyclo [3.3.1.13,7] dec-1-ylamino)carbonyl]amino]-dodecanoic acid), a known EH inhibitor, further supported our claim (Figure 1G). The IC_50_ value (9.4 ± 0.5 nM) of AUDA was determined to test its potency against EphH (Supplementary Figure S3).
ii. *EH, activity with styrene oxide (Red assay)*: As the commercial substrate (PHOME) used in the above assay is sensitive to esterase activity as well; EphH activity was also determined with another EH substrate, styrene oxide. Styrene is a lipophilic compound that is activated to a genotoxic intermediate (styrene oxide), which is rapidly inactivated by EHs (Cirillo et al., 2009). Therefore, it was used as a potential EH substrate. The colorimetric assay was carried out using different concentrations of purified rEphH (10–60 ng/μl) with 100 mM substrate. The reaction with the enzyme added showed degradation in the red color caused by oxidation of adrenaline by NaIO_4_, demonstrating the EH activity of the enzyme, in contrast to the control reaction with no enzyme, which produced a deep red color. The color loss was proportional to the amount of enzyme added. With 60 ng/μl of purified EphH, almost 90% of the red color was lost (Figure 1H; Chownk et al., 2017).

**Table 1:** Michaelis Menten Kinetic parameters of EphH and sEH.

	rEphH	sEH
*K*_m_	21.03 μM	26.2 μM
*V*_max_	1.027 μmol min^−1^	1.08 μmol min^−1^
*K*_cat_/*K*_m_	0.87 s^−1^ μM^−1^	0.115 s^−1^ μM^−1^
IC_50_ of AUDA	9.4 ± 0.5 nM	5.5 ± 0.5 nM

### EphH regulates survival of Mtb in response to acidic pH

**Figure 2:**
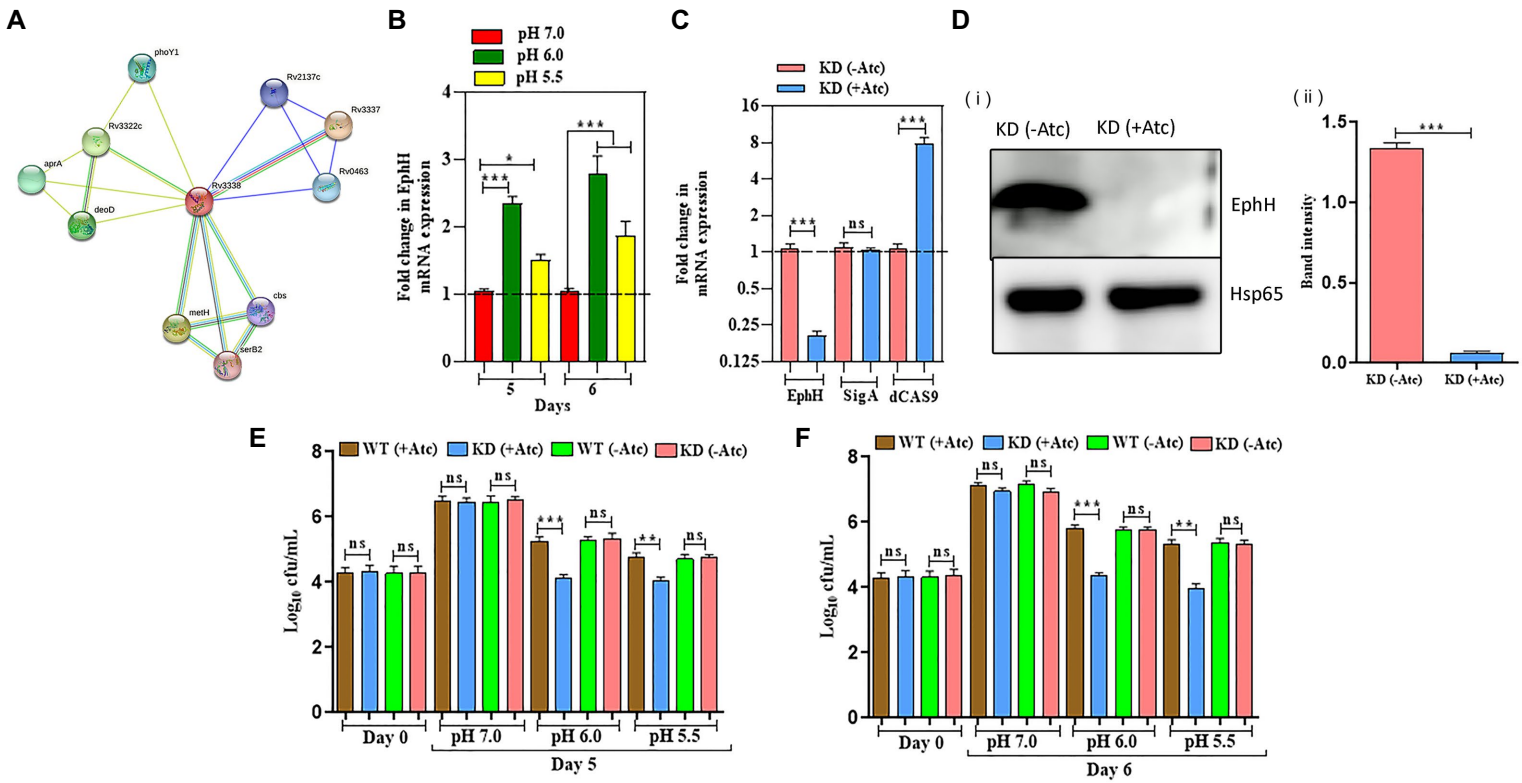
EphH regulates the survival of Mtb in response to acidic pH. **(A)** Shows various Rv3338 interacting proteins; the data was obtained from STRING software. The legend for different colors in the connecting lines. **(B)** Shows the relative expression of EphH transcript in WT Mtb H37Rv under acidic stress. WT Mtb was grown till the log phase (OD_600_ 0.4) and was diluted to OD_600_ 0.05, exposed at pH 7.0, 6.0, and 5.5. The transcript levels were monitored for 6 days. **(C)** EphH KD strain of Mtb was generated by CRISPRi technology. Levels of EphH were examined by qRT-PCR and compared in ATc-treated and untreated cultures of EphH KD. As shown in the figure, ATc treatment resulted in ~92% suppression of EphH, whereas no change was observed in the expression of the control gene sigA. Likewise, dcas9 expression was also analyzed in these cultures to confirm its induction by ATc. **(D)** (i) Construction of EphH KD was also confirmed through western blotting. WT & KD cultures were grown till the log phase (OD_600_ 0.4) and induced with anhydrotetracycline (Atc). (ii) Both cultures were collected the next day and were checked for EphH expression. The blot was quantified by Image J software. **(E,F)** WT and EphH KD cultures were grown till log phase (OD_600_ 0.4), diluted to OD_600_ 0.05, and then exposed to pH 7.0, 6.0, and 5.5. The survival of Mtb was monitored for 5 **(E)** and 6 **(F)** days. Viable cells were estimated through cfu assay. Statistical significance of data, wherever applicable, is indicated by ns: *p* > 0.05; ***p < 0.001. Data plotted are mean ± SD of three independent experiments. Ordinary one-way ANNOVA test was applied for the statical analysis of all the graphs present in the figure. *p > 0.01, **p > 0.05, ***p < 0.001.

As reported earlier, EphH was found to be bioinformatically linked to aprABC locus, whose expression was induced during *in vitro* acidic pH (Abramovitch et al., 2011). To confirm the same, we conducted protein–protein interaction analysis through the STRING software to check its correlation with other related proteins. STRING is a database of known and predicted protein–protein interactions, the interactions come from computational prediction, knowledge transfer across species, and interactions gathered from other (primary) databases. Interactions in STRING are derived from five main sources: Genomic Context Predictions, High-throughput Lab Experiments (Conserved) Co-Expression, automated Text mining, previous knowledge in databases. The data shown in Figure 2A shown its interaction with many Mtb proteins, including aprA which proves that it is associated with aprABC locus. The results obtained paved the way for further experiments. After that, the relative expression of EphH gene were analyzed at pH 6.0 and 5.5 in wild-type H37Rv (WT) till 6 days, to check its expression levels during acid stress as described previously (Vandal et al., 2008). The transcript levels of EphH were upregulated at both pH from the 5th day; however, transcript levels were more upregulated at pH 6.0 compared to pH 5.5 with 0.76-fold induction and a *p* value <0.0001 (Figure 2B). The EphH transcript levels at pH 6.0 and pH 5.5 remain unaffected at early time points till day 4. Together these results demonstrate that EphH expression was upregulated during acid stress in WT Mtb. The EphH protein levels were also analyzed at pH 7.0, 6.0, and 5.5 on the 5th and 6th day (Supplementary Figure S11). The data showed increased EphH expression on the 5th day and 6th day at pH 6.0 and 5.5 in WT Mtb. Next, the effect of EphH was analyzed on the survival of Mtb using EphH knockdown (KD) strain. The KD strain was constructed, and results were validated through qPCR and WB analysis (Figures 2C,Di,ii. Then, the relative expression levels of the EphH transcript were determined in the KD strain for 14 days to check the stability of KD for conducting further survival analysis and growth kinetics (Supplementary Figure S9). Subsequently, the growth kinetics of WT and KD strains was analyzed at different pH values (Supplementary Figures S6–S8) and the bacteria were plated on MB 7H11 agar for enumerating cfu. After knocking down the EphH expression, severe growth defect was observed at pH 6.0 and 5.5 from the 5th day, especially on the 6th day, with a 1.5 log_10_ reduction at pH 6 and 1.3 log_10_ reduction at pH 5.5 (Figures 2E,F). The WT (−Atc) and KD (−Atc) were included as control groups in all the experiments. Taken together, our results showed that EphH plays a major role in the survival of Mtb during *in vitro* acidic stress.

### EphH protects Mtb in response to phagosomal acidification inside macrophages

**Figure 3:**
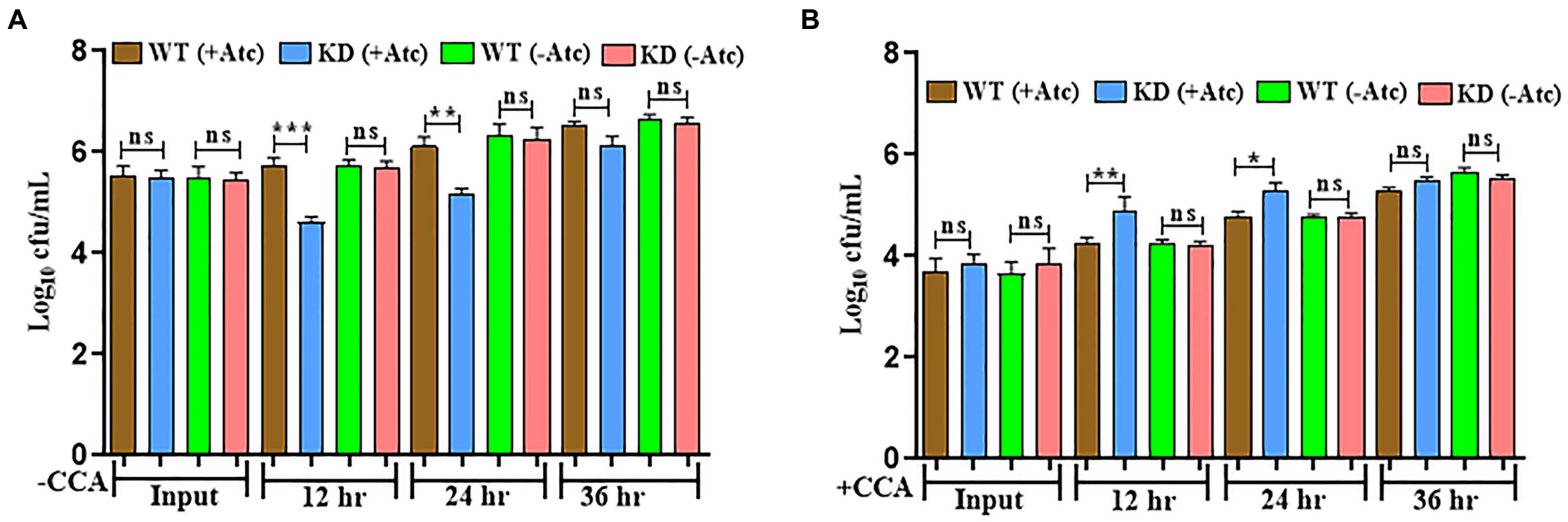
EphH is required for survival of Mtb inside phagosomes and plays a role in response to phagosomal acidification **(A)** J774 A.1 cells were infected with WT and EphH KD strains, bacterial survival was monitored at 12, 24, and 36 h. **(B)** Treatment of J774 A.1 cells was done with CCA or vehicle control (acetonitrile), and then the infection was done with WT and EphH KD strains. Afterwards, at 12, 24, and 36 h, the intraphagosome survival of Mtb was monitored by enumerating cfu. The three individual experiments were conducted in quadruplicate for each data shown. *p* < 0.0001 (CCA-treated EphH KD-infected J774 A.1 phagosomes as compared with untreated control). Statistical significance of data, wherever applicable, is indicated by ns: *p* > 0.05; ***p < 0.001. Data plotted are mean ± SD of three independent experiments. Ordinary one-way ANNOVA test was applied for the statical analysis of all the graphs present in the figure. *p>0.01, **p>0.05, ***p<0.001.

As already stated in previous reports, EphH has been associated with pH-regulated aprABC locus, which plays an important role in macrophage adaptation (Abramovitch et al., 2011). Therefore, the survival of Mtb was monitored inside macrophages. J774A.1 cells were infected with WT & EphH KD strains, and survival was monitored at 12, 24, and 36 h as described earlier (Singh et al., 2017). The result showed a noticeable growth reduction in the EphH KD strain by 1.5 log_10_ and 1.0 log_10_ at 12 h and 24 h, respectively (Figure 3A). There was no significant difference observed between WT and KD groups at 36 h (Figure 3A). After investigating the role of EphH in the survival of Mtb inside phagosomes, we wanted to identify the variable responsible for the observed phenotype. It was hypothesized that the accountable variable could be acidic pH. As already stated, EphH includes the most prominent group of aprA-induced genes, which show differential expression while encountering acidic pH; therefore, the observed phenotype inside macrophages could be a result of acid stress. Therefore, the acidification was restricted inside phagosomes using a specific V-ATPase inhibitor-CCA. V-ATPases are in charge of acidification inside macrophages, these also help in maintaining neutral pH in vacuoles. The results shown increased cfu levels of EphH KD compared to WT at 12 and 24 h with 0.66 log_10_ and 0.5 log_10_ increments (Figure 3B). In all the experiments the WT (−Atc) and KD (−Atc) were included as control groups. The reason behind the observed phenotype could be the intramycobacterial mycothiol redox potential. Previous reports have stated that the limited acidification encountered inside macrophages is sufficient to induce reductive shift in mycothiol redox potential of Mtb during infection (Mehta et al., 2016). Thus, EphH supports Mtb growth during phagosomal acidification phenomena inside the host.

### EphH shows the pH dependant survival of Mtb during nutrient starvation

**Figure 4:**
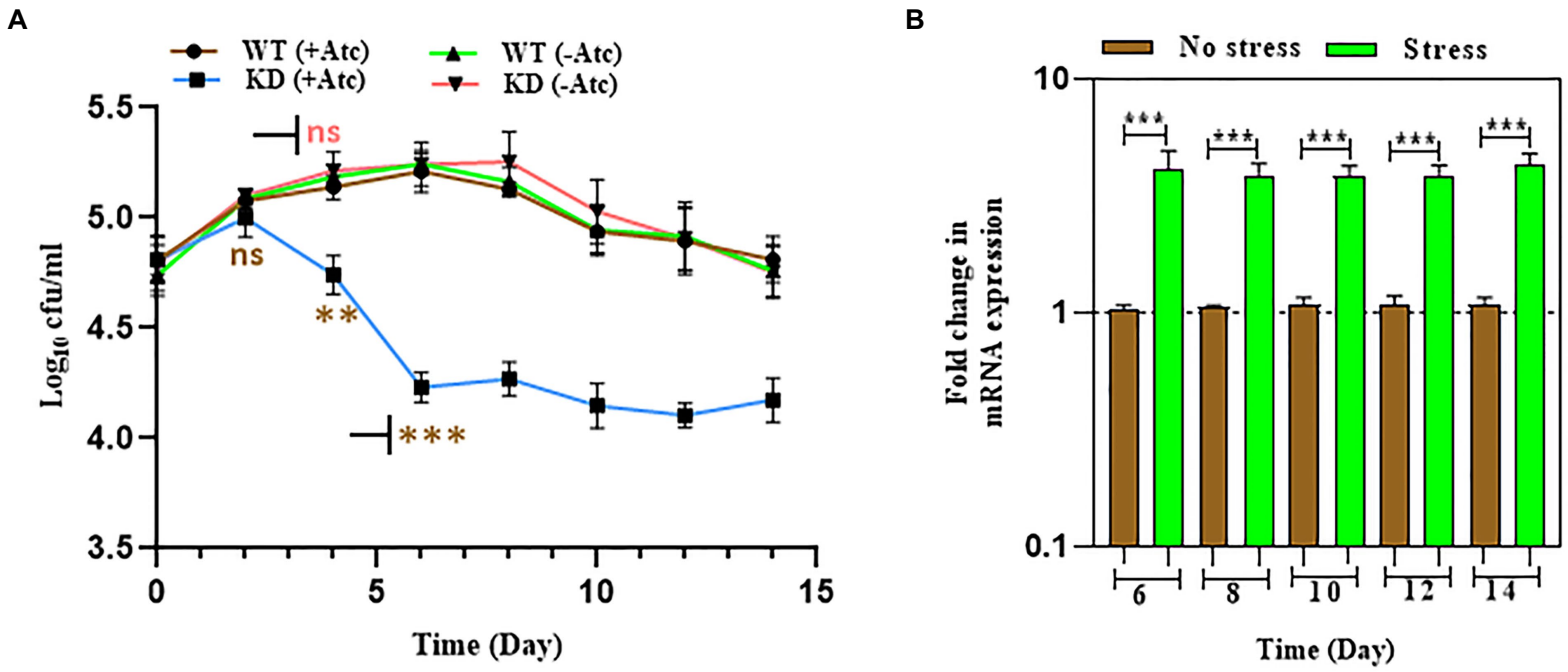
EphH shows pH dependency for the survival of Mtb, but not nutrient dependency **(A)** shows the effect of nutrient starvation stress at pH 7.0 on WT and EphH KD strains. Cultures were grown till log phase (OD_600_ 0.4) and were then diluted to OD_600_ 0.05 and exposed to PBS + 0.05% tween-80 at pH 7.0. cfu determination was done from day 2 to day 14. A significant reduction was seen from the 4th day at pH 7.0. The pH 6.0 and 5.5. groups were also added in the experiments, the data observed shown no change (Supplementary Figures S12A,B). **(B)** Shows the relative expression of EphH transcript under nutrient starvation at pH 7.0 till 14 days. WT culture was grown till log phase (OD_600_ 0.4) and was then diluted to OD_600_ 0.05 and exposed to PBS+ 0.05% tween-80 at pH 7.0 till 14 days. Cultures were collected from day 2 to day 14 and checked for EphH transcript levels. The pH 6.0 and 5.5 groups were also added in the experiments, the data observed shown no change. Data representing transcript levels at pH 6.0 and 5.5 are given in Supplementary Figures S13A,B. Statistical significance of data, wherever applicable, is indicated by ns: *p* > 0.05; ***p < 0.001. Data plotted are mean ± SD of three independent experiments. Two-way ANNOVA analysis was done for **(A)** and ordinary one-way ANNOVA was applied for **(B)**. **p>0.05, ***p<0.001.

Mtb intraphagosome transcriptome is a combined response that considers various signals, including pH, ion concentrations, nutritional stress, and oxidative damage (Schnappinger et al., 2003). Previous studies have reported that acidic stress is more pronounced during nutrient-limited environment (Piddington et al., 2000). So, Mtb survivability was monitored in PBS+ 0.05% tween at pH 7.0, 6.0, and 5.5. Interestingly Mtb growth was severely impaired from the 4^th^ day of the experiment at pH 7 (Figure 4A), but no significant change was observed at pH 6.0 and 5.5 (Supplementary Figures S12A,B). To validate the observed phenotype, the relative expression of EphH was checked in WT Mtb strain at pH 7.0 during nutrient-depleted conditions. The result revealed significant upregulation of EphH from the 6th day (Figure 4B), again no significant change was seen at pH 6.0 and pH 5.5 (Supplementary Figures S13A,B). The above results demonstrate that EphH also supports Mtb growth under the nutrient-deprived condition at pH 7.0 although has pH dependency for the survival of Mtb. WT (−Atc), and KD (−Atc) were included in control groups in the experiments.

### EphH knockdown shows increased ROS generation in acidic stress and nutrient depleted conditions

**Figure 5:**
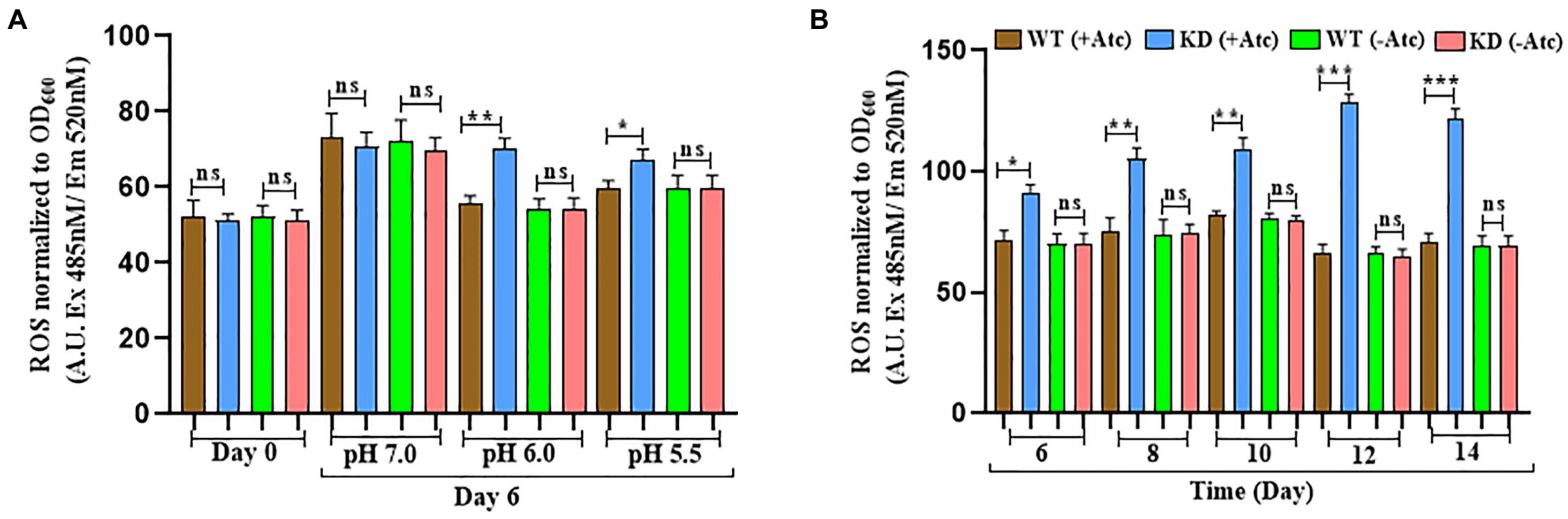
EphH KD shows increased ROS generation in acidic and nutrient starvation conditions. **(A)** Represents the ROS generation in WT and EphH KD strain at pH 7.0, 6.0, and 5.5 on the 6th day. WT and EphH KD strains were grown until the log phase (OD_600_ 0.4) and diluted to OD_600_ 0.05. Subsequently, exposed to pH 7.0, 6.0 and 5.5. ROS generation was monitored for 6 days. **(B)** Represents the ROS generation in nutrient starvation conditions till 14 days. WT and EphH KD strains were grown until the log phase (OD_600_ 0.4) and diluted to OD_600_ 0.05 in PBS+ 0.05% tween-80. ROS generation was monitored for 14 days. Statistical significance of data, wherever applicable, is indicated by ns: *p* > 0.05; ***p < 0.001. Data plotted are mean ± SD of three independent experiments. Ordinary one-way ANNOVA test was applied for the statical analysis of all the graphs present in the figure. *p>0.01, **p>0.05, ***p<0.001.

Previous studies have shown that acidic pH alone leads to accumulation of ROS indicating that acidic pH results in enhanced ROS formation or inhibits oxidative stress resistance mechanisms (Mehta et al., 2016; Coulson et al., 2017). Studies have also reported that Mtb encounters various stresses including nutrient starvation, acidic pH, oxidative and nitrosative stress inside macrophages that results in enhanced ROS levels, therefore the endogenous ROS levels were measured during acid and nutrient depleted stress (Choudhary et al., 2022). During acid stress, the levels of endogenous ROS were upregulated on the 6th day in EphH KD compared to WT with 14.5-fold induction at pH 6.0 and 8-fold induction at pH 5.5, but no significant change was observed at pH 7.0 (Figure 5A).

The endogenous ROS levels were also upregulated during nutrient-depleted conditions in EphH KD compared to WT at pH 7.0. Significant upregulation was seen from the 6th day to the 14th day (Figure 5B). WT (−Atc) and KD (−Atc) were included experimental controls. Altogether, these results demonstrated that EphH plays a role in resistance to endogenous ROS generation while encountering the *in vitro* stresses, and the phenomenon is pH dependent.

### EphH plays an essential role in subverting phagosomal acidification

**Figure 6:**
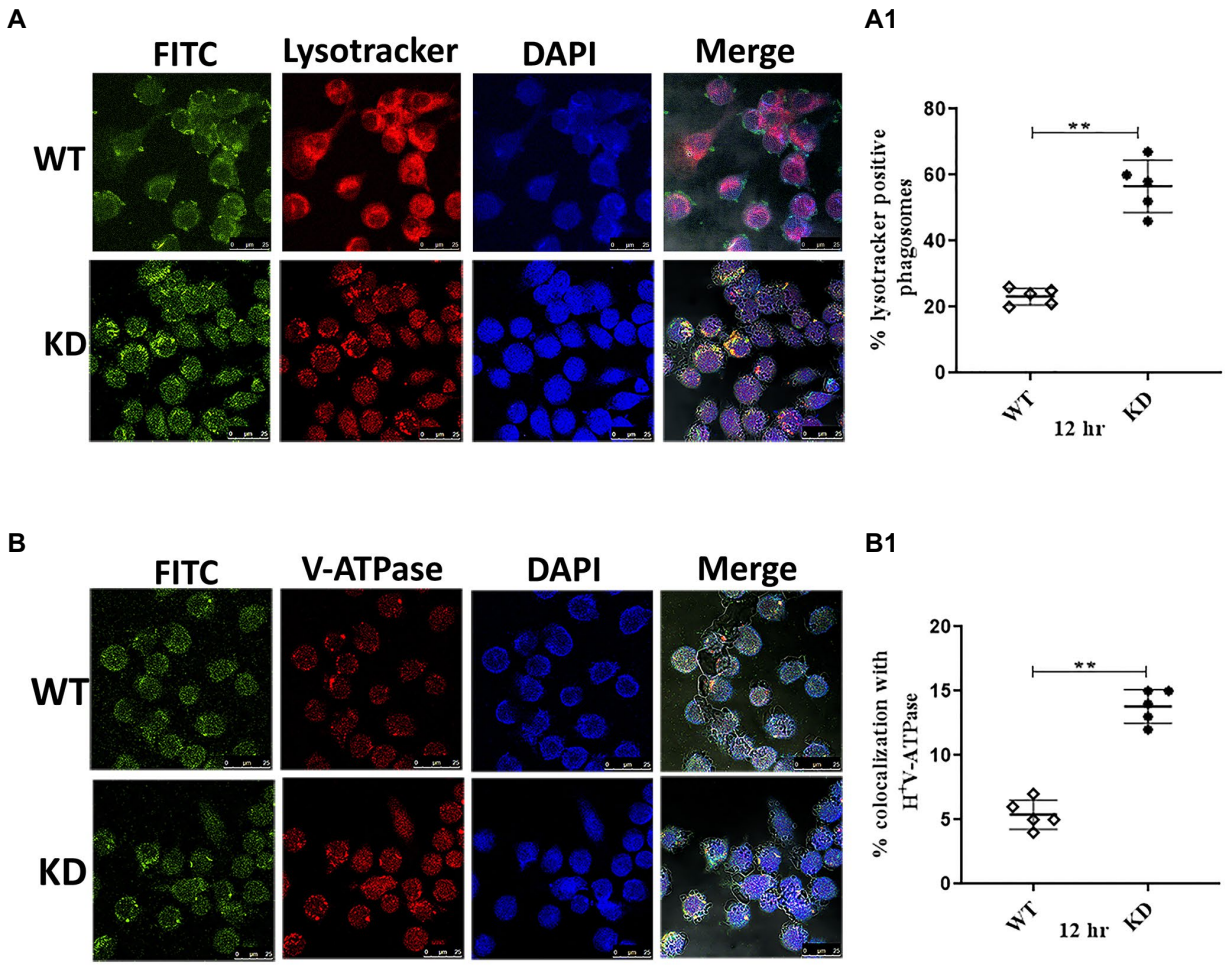
EphH is required to subvert phagosomal acidification-Inside J774A.1 phagosomes, Mtb EphH is augmented in phagosomes positive for lysosomal markers. The infection was done with FITC-labeled WT and KD strains in J774A.1 cells with (MOI of 10). **(A)** Represent staining with lysotracker, **(B)** represent staining with immunofluorescent antibodies, V-ATPase and **(A1,B1)** is the ImageJ quantification of three independent experiments conducted for each group depicted in panels **(A,B)** respectively. In the confocal images, green represents FITC-labeled bacteria; red indicates Lysotracker or V-ATPase; blue indicates DAPI; cell morphology is shown by differential interference contrast (DIC), and yellow represents merged image signals. Scale bar 25 μm (for panels **A,B**). The graphs show the mean values of bacterium-containing phagosomes, which stained positive for markers. Where applicable, the statistical significance of data is indicated by ns: *p* > 0.05. Data plotted are mean ± SD of three independent experiments. Unpaired *t*-test was applied for statical analysis in **(A1,B1)**. **p>0.05.

From the intracellular survival studies, it was clear that EphH plays a role during phagosome acidification. Still, we wanted to get insights as to whether it controls phagosome maturation also. So, the acidification status of EphH KD containing phagosomes was examined. J774A.1 cells were infected with WT, and EphH KD strains at MOI of 10. The studies were conducted 12 h post-infection. The lysotracker red DND-99 staining revealed that WT primarily resided in non-acidified phagosomes compared to EphH KD, which mostly resided in acidified phagosomes. The number of EphH KD bacilli which were found colocalized to acidified phagosomes were (283 bacilli) in comparison to WT (116 bacilli) with a *p*-value <0.0001 (Figure 6A). The phagosome acidification is caused by a molecular proton motor V-ATPase. So, the presence of V-ATPase in acidified phagosomes was examined. Several reports have shown that Mtb actively stops phagosome acidification by averting recruitment or prompting the degradation of V-ATPase (Sturgill-Koszycki et al., 1994, 1996; Clemens and Horwitz, 1995). To test this, the infected phagosomes were immunostained for human V-ATPase, and colocalization was measured. A notably greater percentage of EphH KD containing phagosomes were found to be positive for V-ATPase (69 bacilli) in comparison to WT (27 bacilli). ***p value <0.0001 (Figure 6B). The graphs representing quantitative panels of all three data are shown in Figures 6A1,B1. As phagosomal acidification is an early step in the phagosomal maturation process, the status of KD-containing phagosomes was analyzed for the presence of a late endo-lysosomal fusion marker, CD63. Previously, it has already been reported that Mtb can obstruct phagosomes from maturing into a CD63-positive state (Yates et al., 2005; Carranza and Chavez-Galan, 2019). Our data showed no significant difference between EphH KD-containing CD63 positive phagosomes (21 bacilli) and WT-containing CD63 positive phagosomes (14 bacilli; Supplementary Figure S14).

## Discussion

Mtb, one of the most successful bacterial human pathogens, has developed extensive mechanisms to survive, grow and persist inside the host by overcoming various host–induced stresses like ROS and nutrient depletion combined with acidic pH (Choudhary et al., 2022). Upon entering the phagosome, Mtb encounters a range of pH at different growth stages. At the time of entry in macrophage, the resting macrophage has a pH of 6.2, followed by 4.5–5.5 upon activation (Vandal et al., 2008). During this time, Mtb modulates its genome for survival within the macrophage. Several genes of the Mtb genome have already been predicted to be induced at pH 6 and 5.5 and play a critical role in survival and growth inside macrophages (Vandal et al., 2008). Immediately after the invasion to the resting macrophage, mild acidification in Mtb containing phagosome appears to be a key trigger for transcriptional adaptation. Thus, in early Mtb-macrophage interactions, pH plays a key role as an environmental cue. The present study aimed to characterize the role of EphH, which interestingly was found in previous studies to be associated with the aprABC locus of Mtb. aprABC is an Mtb*-*specific locus whose gene expression is induced during growth inside macrophages in early interaction with Mtb and in *in vitro* acidic environments (Abramovitch et al., 2011). aprABC consists of aprA, aprB and aprC genes. The previous studies show the induced expression of aprA and aprB genes at pH 6.0 and 5.5. Subsequently, microarray studies were conducted to check the differential expression of aprA, aprB, and aprC-induced genes at pH 6.0 and 5.5. Perhaps, EphH was included in the list of the most prominent group of aprA-induced genes spanning a 40.4 kb region between Rv3301c and Rv3338 having a cluster of 21 predicted cistrons. Twenty genes from this region were found to be significantly repressed in the knockout mutant of aprABC, which means they play a crucial role in regulating survival inside macrophages during acid stress (Abramovitch et al., 2011).

EphH is annotated as a conserved hypothetical protein that corresponds to almost 25% of all ORFs present in the 4.4 Mb Mtb genome and is often regarded as an enormous pool of overlooked genes. The bioinformatic analysis revealed that EphH belongs to the alpha/beta hydrolases superfamily containing the AB hydrolase-1 domain and is predicted to be an EH. Epoxide hydrolases are enzymes that metabolize compounds containing an epoxide residue by an epoxide hydrolysis reaction and convert the substrate to two hydroxyl residues to form diol products. Epoxides (3-membered cyclic ethers) are generated in the cells through several metabolic pathways by enzymatically oxidizing xenobiotic compounds. These have highly polarized oxygen–carbon bonds and strained rings, which make them hyper-reactive and unstable in the aqueous environment. The nucleophilic activity can lead to irreversible toxic effects that can cause mutagenic and carcinogenic effects on DNA and proteins (Cirillo et al., 2009). Therefore, the control of epoxide levels within the cell is crucial.

To date, eight EH enzymes have been identified in the Mtb genome (EphA, EphB, EphC, EphD, EphE, EphF, EphG, and MesT). Mtb EHs have been identified as potential therapeutic targets due to their role in mycolic acid metabolism (Johansson et al., 2005). EH enzyme production is not restricted to Mtb; numerous species, including humans, employ these enzymes for various reasons. sEH, which is primarily present in the cytoplasm of hepatocytes, is a major human EH (Madacki et al., 2018). This enzyme’s C-terminal domain is structurally similar to EphB.

Interestingly an analogue of EphB, mel2, which is conserved between *M. marinum* and Mtb, has been identified as a potential drug target as mutations on this locus of *M. marinum* converts it to an attenuated strain unable to infect murine and fish macrophage (El-Etr et al., 2004). Previous studies have reported that EphC expression was upregulated in the absence of an Mtb membrane-bound metalloprotease Rv2869c that regulates cell envelope composition and plays a role in *in vivo* growth of Mtb (El-Etr et al., 2004). Another EH, EphD, is involved in the processing of epoxy mycolates. Overproduction of EphD leads to an accumulation of mycolic acids and a concomitant decrease in epoxy mycolates (El-Etr et al., 2004; Madacki et al., 2021). EphG is known to be involved in detoxification following oxidative damage to lipids (Madacki et al., 2021). Studies conducted with MesT showed it is essential for the optimal growth of Mtb in the presence of styrene oxide (Chownk et al., 2017). The role of EphA, EphE, and EphF remains unexplored. In this context, we have identified a unique EH encoded by Rv3338 by bioinformatic analysis and renamed it EphH after confirming its activity by enzyme assays. Our data demonstrated that the EphH showed significant activity with 50 μM substrate (PHOME). The activity was further confirmed using AUDA, its specific inhibitor. As this commercial substrate is sensitive to esterase activity, the EH activity of purified rEphH was performed with styrene oxide, as reported earlier for Mtb MesT (Chownk et al., 2017). The positive results thus unambiguously established EphH as an EH.

To get insights into the role of EphH during host-pathogen interaction, the expression of EphH was downregulated in a pathogenic slow-growing Mtb H37Rv strain, using the CRISPRi approach as reported by Rock et al. (2017). The downregulation of EphH severely affected the survival of Mtb during acidic stress at pH 6.0 and 5.5. Furthermore, the cfu of Mtb was significantly reduced inside macrophages indicating that EphH plays a role in the survival of Mtb during infection to the host. Following this, the variable behind the observed phenotype was to be investigated. As stated above, EphH involved the most vital group of aprA-induced genes between Rv3301c and Rv3338. As aprA expression was induced at acidic pH, we hypothesized that the accountable variable for the phenotype observed in macrophages could be acidic pH. To test this hypothesis, the role of EphH was studied in response to phagosomal acidification inside macrophages. For this purpose, the acidification was blocked inside macrophages using V-ATPase inhibitor-CCA. V-ATPases are the proton pumps responsible for acidification inside macrophages. The speculation was that if acidic pH was the responsible variable, the observed phenotype should be reverted. As expected, the data obtained show a dramatic increment of Mtb growth in response to restricted acidification inside phagosome. The reason behind the observed phenotype could be intramycobacterial mycothiol redox potential. Previous studies have reported that phagosomal acidification affects intramycobacterial mycothiol redox potential. Further, the studies have shown that treatment with V-ATPase inhibitors (CCA and BafilomycinA1) in macrophages infected with WT Mtb results in declination of the proportion of bacilli having reduced mycothiol redox potential as compared with untreated macrophages post infection. This indicates that cells express nearly neutral pH in vacuoles upon V-ATPase inhibitor treatment. The limited acidification encountered inside macrophages is sufficient to induce reductive shift in mycothiol redox potential of Mtb during infection (Mehta et al., 2016).

One of the vital processes by which Mtb resists acid stress is arresting the routine phagosome maturation process. In this regard, we wanted to check whether EphH is involved during the initial phagosomal acidification process or at the later maturation stage. To test this, the acidification status of phagosomes containing EphH KD was analyzed with lysotracker, V-ATPase and CD-63. The confocal results showed a higher percentage of EphH KD remained in acidified phagosomes and were positive for V-ATPase, but no significant data could be obtained for CD-63. Altogether, the results established that EphH had a role in initial phagosomal acidification but not at the later maturation stage.

The role of EphH was explored during nutrient starvation stress, as several reports showed that nutrient-limited environments make acidic pH-induced stress more severe (Piddington et al., 2000). However, our results did not show any significant effect on the survival of Mtb at pH 6.0 and 5.5. Surprisingly, a significant survival defect was seen at pH 7.0, indicating that the role of EphH in the survival of Mtb is pH-dependent but not nutrient-dependent.

Previous studies have shown that acidic pH affects intracellular redox status, creating a reducing environment in the cytoplasm of Mtb and, in the absence of the pH-regulated TCS PhoPR, further reducing the cytoplasm at acidic pH, demonstrating the existence of pH-dependent mechanisms to maintain redox homeostasis. Another study reporting the compound AC2P36 that selectively kills Mtb at acidic pH shows that the compound, in combination with acidic pH, doubles the accumulation of intracellular ROS compared with cells at neutral pH (Mehta et al., 2016; Coulson et al., 2017). Studies have also reported that Mtb experiences various stresses inside macrophages, including low pH and nutrient stress, which leads to enhanced ROS levels (Choudhary et al., 2022). Therefore, the endogenous ROS levels were analyzed during *in vitro* stress. Our results revealed significantly higher endogenous ROS levels in EphH KD compared to WT during both acidic stress and nutrient-depleted conditions. The data suggested that EphH might play a critical role for Mtb in providing resistance to ROS generation during *in vitro* stress environment.

Taken together, the results of our study established EphH as a unique EH and demonstrated its role in pathogen’s survival during *in vitro* acid stress conditions, nutrient depletion, and inside macrophages.

The study also demonstrated that the role of EphH is pH regulated but not nutrient-dependent. The result also showed EphH as a critical player in the survival of Mtb during phagosome acidification and in providing resistance to endogenous ROS generation during *in vitro* stress conditions. Thus, our findings have led to the discovery of an EH which plays a role in the survival and growth of Mtb inside the host. This study to the best of our knowledge the first report defining the role of Mtb EH in phagosomal acidification, compared to previous ones, which are known to play the role only in mycolic acid metabolism, indicating that EphH could be a potential therapeutic target.

## Data availability statement

The raw data supporting the conclusions of this article will be made available by the authors, without undue reservation.

## Author contributions

TG, SD, UG, and AD designed the experiments. TG, SD, SS, MI, and AM performed the experiments. TG, SD, UG, SC, and AD analyzed the data. TG, SC, and AD wrote the manuscript draft. All authors contributed to the article and approved the submitted version.

## Funding

The study received financial support from DST Women Scientist-A project GAP0327 (File No. SR/WOS-A/LS-361/2018). TG thanks DST (Government of India), SD, SS, and MI to thank CSIR and AM thanks UGC for their fellowship.

## Conflict of interest

The authors declare that the research was conducted in the absence of any commercial or financial relationships that could be construed as a potential conflict of interest.

## Publisher’s note

All claims expressed in this article are solely those of the authors and do not necessarily represent those of their affiliated organizations, or those of the publisher, the editors and the reviewers. Any product that may be evaluated in this article, or claim that may be made by its manufacturer, is not guaranteed or endorsed by the publisher.

## References

[ref1] Abramovitch R. B. Rohde K. H. Hsu F. F. Russell D. G. (2011). aprABC: a *Mycobacterium tuberculosis* complex-specific locus that modulates pH-driven adaptation to the macrophage phagosome. Mol. Microbiol. 80, 678–694. doi: 10.1111/j.1365-2958.2011.07601.x, PMID: 21401735 PMC3138066

[ref2] Armstrong J. A. Hart P. D. A. (1971). Response of cultured macrophages to *Mycobacterium tuberculosis*, with observations on fusion of lysosomes with macrophages. J. Exp. Med. 134, 713–740. doi: 10.1084/jem.134.3.713, PMID: 15776571 PMC2139093

[ref3] Biswal B. K. Morisseau C. Garen G. Cherney M. M. Garen C. Niu C.. (2008). The molecular structure of epoxide hydrolase B from *Mycobacterium tuberculosis* and its complex with a urea-based inhibitor. J. Mol. Biol. 381, 897–912. doi: 10.1016/j.jmb.2008.06.030, PMID: 18585390 PMC2866126

[ref4] Carranza C. Chavez-Galan L. (2019). Several routes to the same destination: inhibition of phagosome-lysosome fusion by *Mycobacterium tuberculosis*. Am J Med Sci 357, 184–194. doi: 10.1016/j.amjms.2018.12.003, PMID: 30797501

[ref5] Choudhary E. Sharma R. Pal P. Agarwal N. (2022). Deciphering the proteomic landscape of *Mycobacterium tuberculosis* in response to acid and oxidative stresses. ACS Omega. 7, 26749–26766. doi: 10.1021/acsomega.2c03092, PMID: 35936415 PMC9352160

[ref6] Chownk M. Sharma A. Singh K. Kaur J. (2017). mes T, a unique epoxide hydrolase, is essential for optimal growth of *Mycobacterium tuberculosis* in the presence of styrene oxide. Future Microbiol. 12, 527–546. doi: 10.2217/fmb-2016-0206 28492351

[ref7] Cirillo S. L. Subbian S. Chen B. Weisbrod T. R. Jacobs W. R. Jr. Cirillo J. D. (2009). Protection of *Mycobacterium tuberculosis* from reactive oxygen species conferred by the mel2 locus impacts persistence and dissemination. Infect. Immun. 77, 2557–2567. doi: 10.1128/IAI.01481-08, PMID: 19349422 PMC2687327

[ref8] Clemens D. L. Horwitz M. A. (1995). Characterization of the *Mycobacterium tuberculosis* phagosome and evidence that phagosomal maturation is inhibited. J. Exp. Med. 181, 257–270. doi: 10.1084/jem.181.1.257, PMID: 7807006 PMC2191842

[ref9] Cole S. Brosch R. Parkhill J. Garnier T. Churcher C. Harris D.. (1998). Deciphering the biology of *Mycobacterium tuberculosis* from the complete genome sequence. Nature 396:190. doi: 10.1038/24206 9634230

[ref10] Coulson G. B. Johnson B. K. Zheng H. Colvin C. J. Fillinger R. J. Haiderer E. R.. (2017). Targeting *Mycobacterium tuberculosis* sensitivity to thiol stress at acidic pH kills the bacterium and potentiates antibiotics. Cell Chem. Biol. 24, 993.e4–1004.e4. doi: 10.1016/j.chembiol.2017.06.018 28781126 PMC5562523

[ref11] Doerks T. Van Noort V. Minguez P. Bork P. (2012). Annotation of the *M. tuberculosis* hypothetical orfeome: adding functional information to more than half of the uncharacterized proteins. PLoS One 7:e34302. doi: 10.1371/journal.pone.0034302, PMID: 22485162 PMC3317503

[ref12] Ehrt S. Schnappinger D. (2009). Mycobacterial survival strategies in the phagosome: defence against host stresses. Cell. Microbiol. 11, 1170–1178. doi: 10.1111/j.1462-5822.2009.01335.x, PMID: 19438516 PMC3170014

[ref13] El-Etr S. H. Subbian S. Cirillo S. L. Cirillo J. D. (2004). Identification of two *Mycobacterium marinum* loci that affect interactions with macrophages. Infect. Immun. 72, 6902–6913. doi: 10.1128/IAI.72.12.6902-6913.2004 15557611 PMC529147

[ref14] Hart P. D. A. Armstrong J. A. (1974). Strain virulence and the lysosomal response in macrophages infected with *Mycobacterium tuberculosis*. Infect. Immun. 10, 742–746. doi: 10.1128/iai.10.4.742-746.1974, PMID: 4214780 PMC423015

[ref15] Johansson P. Unge T. Cronin A. Arand M. Bergfors T. Jones T. A.. (2005). Structure of an atypical epoxide hydrolase from *Mycobacterium tuberculosis* gives insights into its function. J. Mol. Biol. 351, 1048–1056. doi: 10.1016/j.jmb.2005.06.055, PMID: 16051262

[ref16] Livak K. J. Schmittgen T. D. (2001). Analysis of relative gene expression data using real-time quantitative PCR and the 2− ΔΔCT method. Methods 25, 402–408. doi: 10.1006/meth.2001.1262 11846609

[ref17] MacMicking J. D. Taylor G. A. McKinney J. D. (2003). Immune control of tuberculosis by IFN-γ-inducible LRG-47. Science 302, 654–659. doi: 10.1126/science.1088063, PMID: 14576437

[ref18] Madacki J. Kopál M. Jackson M. Korduláková J. (2021). Mycobacterial epoxide hydrolase EphD is inhibited by urea and Thiourea derivatives. Int. J. Mol. Sci. 22:2884. doi: 10.3390/ijms22062884, PMID: 33809178 PMC7998700

[ref19] Madacki J. Laval F. Grzegorzewicz A. Lemassu A. Záhorszká M. Arand M.. (2018). Impact of the epoxide hydrolase EphD on the metabolism of mycolic acids in mycobacteria. J. Biol. Chem. 293, 5172–5184. doi: 10.1074/jbc.RA117.000246, PMID: 29472294 PMC5892587

[ref20] Mazandu G. K. Mulder N. J. (2012). Function prediction and analysis of *Mycobacterium tuberculosis* hypothetical proteins. Int. J. Mol. Sci. 13, 7283–7302. doi: 10.3390/ijms13067283, PMID: 22837694 PMC3397526

[ref21] Mehta M. Rajmani R. S. Singh A. (2016). *Mycobacterium tuberculosis* WhiB3 responds to vacuolar pH-induced changes in mycothiol redox potential to modulate phagosomal maturation and virulence. J. Biol. Chem. 291, 2888–2903. doi: 10.1074/jbc.M115.684597, PMID: 26637353 PMC4742752

[ref22] Piddington D. L. Kashkouli A. Buchmeier N. A. (2000). Growth of *Mycobacterium tuberculosis* in a defined medium is very restricted by acid pH and Mg2+ levels. Infect. Immun. 68, 4518–4522. doi: 10.1128/IAI.68.8.4518-4522.2000 10899850 PMC98362

[ref23] Rastogi S. Agarwal P. Krishnan M. Y. (2016). Use of an adipocyte model to study the transcriptional adaptation of *Mycobacterium tuberculosis* to store and degrade host fat. Int. J. Mycobacteriol. 5, 92–98. doi: 10.1016/j.ijmyco.2015.10.003, PMID: 26927997

[ref24] Rock J. M. Hopkins F. F. Chavez A. Diallo M. Chase M. R. Gerrick E. R.. (2017). Programmable transcriptional repression in mycobacteria using an orthogonal CRISPR interference platform. Nat. Microbiol. 2:16274. doi: 10.1038/nmicrobiol.2016.274, PMID: 28165460 PMC5302332

[ref25] Rohde K. H. Abramovitch R. B. Russell D. G. (2007). *Mycobacterium tuberculosis* invasion of macrophages: linking bacterial gene expression to environmental cues. Cell Host Microbe 2, 352–364. doi: 10.1016/j.chom.2007.09.006, PMID: 18005756

[ref26] Rohde K. H. Veiga D. F. Caldwell S. Balázsi G. Russell D. G. (2012). Linking the transcriptional profiles and the physiological states of *Mycobacterium tuberculosis* during an extended intracellular infection. PLoS Pathog. 8:e1002769. doi: 10.1371/journal.ppat.1002769, PMID: 22737072 PMC3380936

[ref27] Russell D. G. (2001). *Mycobacterium tuberculosis*: here today, and here tomorrow. Nat. Rev. Mol. Cell Biol. 2, 569–578. doi: 10.1038/35085034, PMID: 11483990

[ref28] Schaible U. E. Sturgill-Koszycki S. Schlesinger P. H. Russell D. G. (1998). Cytokine activation leads to acidification and increases maturation of *Mycobacterium avium*-containing macrophages in murine macrophages. J. Immunol. 160, 1290–1296. 9570546

[ref29] Schnappinger D. Ehrt S. Voskuil M. I. Liu Y. Mangan J. A. Monahan I. M.. (2003). Transcriptional adaptation of *Mycobacterium tuberculosis* within macrophages: insights into the phagosomal environment. J. Exp. Med. 198, 693–704. doi: 10.1084/jem.20030846, PMID: 12953091 PMC2194186

[ref30] Schulz E. C. Henderson S. R. Illarionov B. Crosskey T. Southall S. M. Krichel B.. (2020). The crystal structure of mycobacterial epoxide hydrolase A. Sci. Rep. 10, 1–14. doi: 10.1038/s41598-020-73452-y 33024154 PMC7538969

[ref31] Singh K. H. Jha B. Dwivedy A. Choudhary E. Arpitha G. N. Ashraf A.. (2017). Characterization of a secretory hydrolase from *Mycobacterium tuberculosis* sheds critical insight into host lipid utilization by *M. tuberculosis*. J. Biol. Chem. 292, 11326–11335. doi: 10.1074/jbc.M117.794297, PMID: 28515317 PMC5500798

[ref32] Sturgill-Koszycki S. Schaible U. E. Russell D. G. (1996). *Mycobacterium*-containing macrophages are accessible to early endosomes and reflect a transitional state in normal macrophage biogenesis. EMBO J. 15, 6960–6968. doi: 10.1002/j.1460-2075.1996.tb01088.x, PMID: 9003772 PMC452522

[ref33] Sturgill-Koszycki S. Schlesinger P. H. Chakraborty P. Haddix P. L. Collins H. L. Fok A. K.. (1994). Lack of acidification in *Mycobacterium* macrophages produced by exclusion of the vesicular proton-ATPase. Science 263, 678–681. doi: 10.1126/science.8303277, PMID: 8303277

[ref34] Sullivan J. T. Young E. F. McCann J. R. Braunstein M. (2012). The *Mycobacterium tuberculosis* SecA2 system subverts phagosome maturation to promote growth in macrophages. Infect. Immun. 80, 996–1006. doi: 10.1128/IAI.05987-11, PMID: 22215736 PMC3294638

[ref35] Vandal O. H. Nathan C. F. Ehrt S. (2009). Acid resistance in *Mycobacterium tuberculosis*. J. Bacteriol. 191, 4714–4721. doi: 10.1128/JB.00305-09, PMID: 19465648 PMC2715723

[ref36] Vandal O. H. Pierini L. M. Schnappinger D. Nathan C. F. Ehrt S. (2008). A membrane protein preserves intrabacterial pH in intraphagosomal *Mycobacterium tuberculosis*. Nat. Med. 14, 849–854. doi: 10.1038/nm.1795, PMID: 18641659 PMC2538620

[ref37] Via L. E. Fratti R. A. McFalone M. Pagan-Ramos E. Deretic D. Deretic V. (1998). Effects of cytokines on mycobacterial phagosome maturation. J. Cell Sci. 111, 897–905. doi: 10.1242/jcs.111.7.897 9490634

[ref38] Wolf N. M. Morisseau C. Jones P. D. Hock B. Hammock B. D. (2006). Development of a high-throughput screen for soluble epoxide hydrolase inhibition. Anal. Biochem. 355, 71–80. doi: 10.1016/j.ab.2006.04.045, PMID: 16729954 PMC1964503

[ref39] World Health Organization (2022). Global tuberculosis report 2022.

[ref40] Yang Z. Zeng X. Tsui S. K. W. (2019). Investigating function roles of hypothetical proteins encoded by the *Mycobacterium tuberculosis* H37Rv genome. BMC Genomics 20:394. doi: 10.1186/s12864-019-5746-6 31113361 PMC6528289

[ref41] Yates R. M. Hermetter A. Russell D. G. (2005). The kinetics of phagosome maturation as a function of phagosome/lysosome fusion and acquisition of hydrolytic activity. Traffic 6, 413–420. doi: 10.1111/j.1600-0854.2005.00284.x, PMID: 15813751

