# Supplementary material for: EphH, a unique epoxide hydrolase encoded by Rv3338 is involved in the survival of Mycobacterium tuberculosis under in vitro stress and vacuolar pH-induced changes
Source: Front Microbiol. 2023 Jan 26;13:1092131. doi: 10.3389/fmicb.2022.1092131 (PMC9908614; doi:10.3389/fmicb.2022.1092131)
Supplement: Supplementary file 1 [file Table_1.DOCX]

**Supplementary Information-**

**Figure S1:**

1. Rv3338 overexpressed recombinant protein from E. coli whole cell lysate probed with monoclonal His- tag antibody.
2. Rv3338 recombinant purified protein probed with monoclonal His- tag antibody.

**Figure S2:**


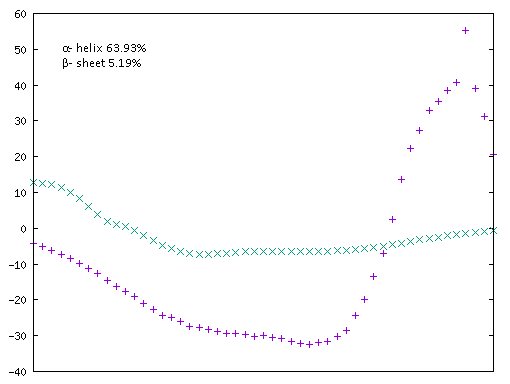

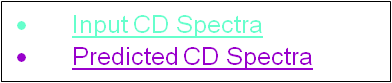


**Figure S3:**


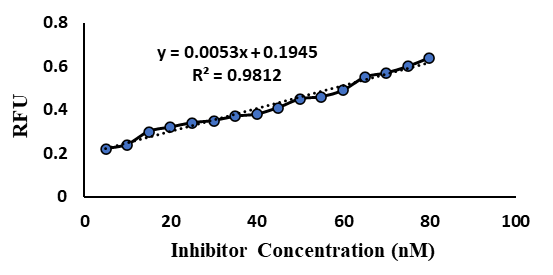


**Figure S4:**

**A**- H37Rv Whole Cell Lysate

**B**- Recombinant purified Rv3338 (positive control) tagged with monoclonal Hsp65 antibody.

**C**- J774 cell lysate (negative control) tagged with monoclonal β- actin antibody.

**Figure S5:**

**Figure S6:**

**Figure S7:**

**Figure S8:**

**Figure S9:**

**B.**

**Figure S10:**

**Figure S11:**


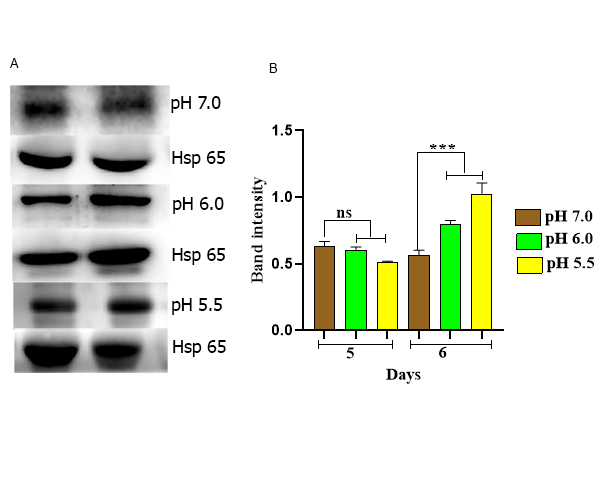


**Figure S12:**

**A.**

**B.**

**Figure S13:**

**A.**

**B.**

**Figure S14:**

**
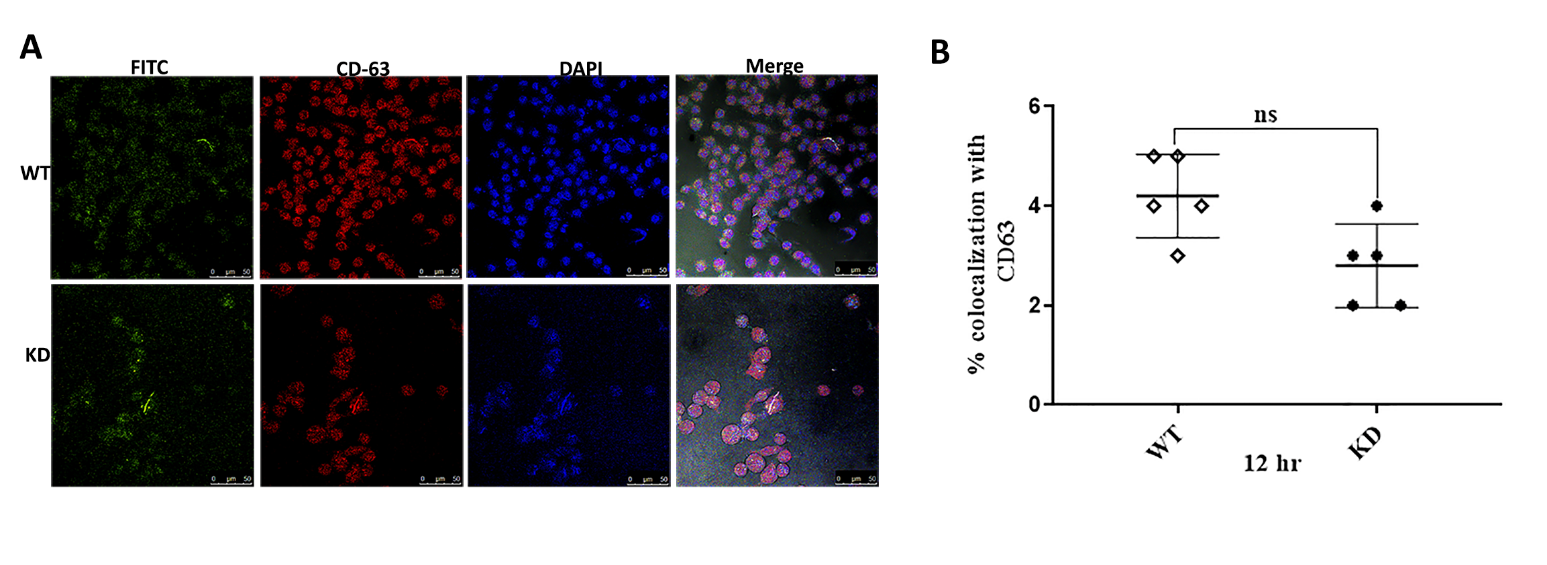
**

**Figure S15:**

**Table S1**

**Primers used for cloning Rv3338 in pET28a**

| Nde I F | GGACCATATGTTGTCCTCGGCGGTG |
| --- | --- |
| Xho II R | CCGCTCGAGCTAACTGGCCCGGACATG |

**Table S2**

**sgRNAs used for constructing Rv3338 CRISPRi Knock down in *M. tuberculosis*-**

| PAM sequence used for Rv3338 knockdown | 3’TAAGAACCGGTTGCCCGCGAAGCCGACG 5’ – 216.2 |
| --- | --- |
| sgRNA used | GCAGCCGAAGCGCCCGTTGGC |
| Rv3338 Forward oligo | GGGAGCAGCCGAAGCGCCCGTTGGC |
| Rv3338 Reverse oligo | AAACGCCAACGGGCGCTTCGGCTGC |

**Primer used to confirm Knock down strain-**

| TTCCTGTGAAGAGCCATTGATAATG |
| --- |

**Table S3**

| qPCR Rv3338 Forward | TGGGAAACCAGCCATATCGT |
| --- | --- |
| qPCR Rv3338 Reverse | ACTTCGAACTTGACCGGACT |
| qPCR dcas9 Forward | GCTACCTGGAGATCCTGACC |
| qPCR dcas9 Reverse | GGATGCCGAAGATGTTGTCC |
| qPCR SigA Forward | AAGACCACGAAGACCTCGAA |
| qPCR SigA Reverse | CGACTCGTCTTCATCCCAGA |
| qPCR Rv3338 Forward | GGCCGCTTCGAGGTATTT |
| qPCR Rv3338 Reverse | CCGACGATATGGCTGGTTT |

**SUPPLEMENTARY FIGURE LEGENDS-**

**Figure S1:** Immunoblotting with anti-His tag with purified recombinant Rv3338. The two bands show the two different batches of eluted protein.

**Figure S2:** Data showing CD data analysis using K2D3 software.

**Figure S3:** Graph representing IC_50_ value of Rv3338 recombinant enzyme.

**Figure S4:** The *M. tuberculosis* whole cell lysate was prepared and used for antibody specificity testing. The different dilutions of Rv3338 antiserum were used (1:2000, 1:1000, 1:500).

**Figure S5:** The figure shows the relative expression of EphH gene in WT from day 1 to day6. The WT strain was grown at OD_600_ 0.05 to 0.9 and the cultures were collected each day and checked for transcript levels. The data shows that EphH expression does not have growth phase dependency.

**Figure S6:** Growth curve analysis of WT EphH KD strains and at pH 7.0.

**Figure S7:** Growth curve analysis of WT EphH KD strains and at pH 6.0.

**Figure S8:** Growth curve analysis of WT EphH KD strains and at pH 5.5.

**Figure S9:** qPCR analysis to check the stability of EphH KD till 14 days.

1. WT and KD groups treated with Atc.
2. WT and KD groups non-treated with Atc.

Statistical significance of data wherever applicable is indicated by ns: p > 0.05; ***p < 0.001. Data plotted are mean ± SD of three independent experiments.

**Figure S10:** The figure shows the relative expression levels of EphH gene from day 1 to day 4 at pH 7.0, 6.0 and 5.5 in WT Mtb.

**Figure S11:** Western blotting results showing expression of EphH at 5^th^ and 6^th^ day respectively at pH 7.0, pH 6.0, pH 5.5.

Statistical significance of data wherever applicable is indicated by ns: p > 0.05; ***p < 0.001. Data plotted are mean ± SD of three independent experiments.

**Figure S12:**

**A and B-**

Figure shows the effect of nutrient depletion during acid stress (pH 6.0 and pH 5.5) on WT and EphH KD strains. Cultures were grown till log phase (OD_600_ 0.4) and then were diluted to 0.05 exposed to PBS+ 0.05% tween-80 at pH 6.0 and pH 5.5. CFU determination was done for both groups till 14 days after every 48 hrs. No significant change was observed through the CFU estimation.

Statistical significance of data wherever applicable is indicated by ns: p > 0.05; ***p < 0.001. Data plotted are mean ± SD of three independent experiments.

**Figure S13:**

**A and B-**

Figure shows the effect of nutrient depletion during acid stress (pH 6.0 and pH 5.5) on EphH expression. WT culture was grown till log phase (OD_600_ 0.4) and then were diluted to 0.05, exposed to PBS+ 0.05% tween-80 at pH 6.0 and pH 5.5. EphH transcript levels were checked till 14 days after every 48 hrs. No significant change was observed till 14 days at both pH 6.0 and pH 5.5.

Statistical significance of data wherever applicable is indicated by ns: p > 0.05; ***p < 0.001. Data plotted are mean ± SD of three independent experiments.

**Figure S14:**

1. The infection was done with FITC-labelled WT and KD strains in J774A.1 cells with (MOI of 10) and cells were stained with immunofluorescent CD-63 antibody. Scale bar is 50 µm for the panel represented.
2. The number of colocalised bacilli were quantified by ImageJ software. The data depicted in the graph represent the quantification of three independent experiments conducted for each group. Scale bar for the panel is 50 µm Statistical significance of data wherever applicable is indicated by ns: p > 0.05; ***p < 0.001. Data plotted are mean ± SD of three independent experiments.

**Figure S15:**

Survival of WT and KD was monitored inside macrophages in presence of 1 nM CCA at 12 hr, 24 hr and 36 hr respectively. The results obtained were same as observed with 10 nM CCA in Figure 3B.
